# Outcomes following upfront radiation versus monitoring in atypical meningiomas: 16-year experience at a tertiary medical center

**DOI:** 10.1093/noajnl/vdab094

**Published:** 2021-06-29

**Authors:** Peter C Pan, David J Pisapia, Rohan Ramakrishna, Theodore H Schwartz, Susan C Pannullo, Jonathan P S Knisely, Gloria C Chiang, Jana Ivanidze, Philip E Stieg, Benjamin Liechty, Andrew Brandmaier, Howard A Fine, Rajiv S Magge

**Affiliations:** 1 Brain and Spine Center, Weill Cornell Medicine, New York, New York, USA; 2 Department of Radiation-Oncology, Weill Cornell Medicine, New York, New York, USA; 3 Department of Radiology, Weill Cornell Medicine, New York, New York, USA; 4 Department of Pathology, Weill Cornell Medicine, New York, New York, USA

**Keywords:** atypical meningioma, overall survival, progression-free survival, radiotherapy, stereotactic radiosurgery

## Abstract

**Background:**

The role of postoperative upfront radiotherapy (RT) in the management of gross totally resected atypical meningiomas remains unclear. This single-center retrospective review of newly diagnosed histologically confirmed cases of World Health Organization (WHO) Grade II atypical meningioma at Weill Cornell Medicine from 2004 to 2020 aims to compare overall survival (OS) and progression-free survival (PFS) of postoperative upfront RT versus observation, stratified by resection status (gross total resection [GTR] vs subtotal resection [STR]).

**Methods:**

Ninety cases of atypical meningioma were reviewed (56% women; median age 61 years; median follow-up 41 months).

**Results:**

In patients with GTR, hazard ratio (HR) of PFS was 0.09 for postoperative upfront RT versus observation alone (95% confidence interval [CI] 0.01–0.68; *P* = .02), though HR for OS was not significant (HR 0.46; 95% CI 0.05–4.45; *P* = .5). With RT, PFS was 100% at 12 and 36 months (compared to 84% and 63%, respectively, with observation); OS at 36 months (OS36) was 100% (compared to 94% with observation). In patients with STR, though PFS at 36 months was higher for RT arm versus observation (84% vs 74%), OS36 was 100% in both arms. HR was not significant (HR 0.76; 95% CI 0.16–3.5; *P* = .73).

**Conclusions:**

This retrospective study suggests postoperative upfront RT following GTR of atypical meningioma is associated with improved PFS compared to observation. Further studies are required to draw conclusions about OS.

**Key Points**

- Upfront RT is associated with improved PFS in new atypical meningioma after GTR.
- Effect of upfront RT on OS in new atypical meningioma after GTR is less clear.
- Retrospective study of atypical meningioma is challenged by need for long follow-up.

**Importance of the Study**

Timing of radiotherapy after resection of newly diagnosed atypical meningioma remains controversial, particularly in the gross total resected tumors. Early postoperative radiotherapy appears to be useful in prolonging time to recurrence, but the effect on overall survival and optimal timing for radiotherapy remains an open question. This study summarizes over 15 years of retrospective experience with newly diagnosed atypical meningioma at a tertiary center. The data confirm that early postoperative radiotherapy improves time to progression in newly diagnosed atypical meningiomas that are gross total resected. The effect on overall survival was less clear. Patient retention is identified as an important barrier to following of long-term outcomes.

Meningioma is the most common adult primary central nervous system tumor.^1^ The World Health Organization (WHO) categorizes meningiomas into 3 grades of increasing biologic aggressiveness—WHO Grade I meningioma, WHO Grade II atypical meningioma, and WHO Grade III anaplastic/malignant meningioma.^2^ Despite advances in our understanding of the genetic landscape of meningioma, which have led to revisions of the histopathologic criteria for diagnosing atypical or anaplastic meningioma,^3–8^ systemic treatments remain limited and management primarily depends on surgical intervention and radiotherapy (RT). Fortunately, the majority of meningiomas are slow-growing WHO Grade I tumors. These have an indolent clinical course and can oftentimes be managed expectantly, with resection reserved for cases where consideration of age, symptoms, operative risk, and medical comorbidities favors intervention, especially in the setting of growing or symptomatic tumors. The extent of operation is broadly divided into gross total resection (GTR) or subtotal resection (STR)—classically judged by surgical impression, the modern inclusion of postoperative magnetic resonance image (MRI) can help with accuracy. Extent of resection is graded by the Simpson criteria, which takes into consideration the treatment of the dural attachment.^9^ With a complete resection, a WHO Grade I meningioma often requires no further treatment and clinically can be considered cured.

The optimal management of atypical meningiomas remains a topic of active investigation. WHO Grade II atypical meningioma is currently histologically defined by 4–19 mitotic figures per 10 high powered fields (with a newer threshold of 6 mitotic figures being considered), brain invasion, or at least 3 of the following histologic features—increased cellularity, small cells with a high nucleus-to-cytoplasm ratio, prominent nucleoli, sheet-like or pattern-less growth, or geographic necrosis. Atypical meningiomas follow a more active clinical course and require closer follow-up, as they can grow more quickly, cause more symptoms, and have higher rates of recurrence than WHO Grade I meningioma.^10^ Extent of resection for atypical meningiomas correlates with rates of local control.^11–14^ Postoperative adjuvant radiation is often delivered with the goal of prolonging time to tumor recurrence, particularly in the setting of STR, and can be in the form of either fractionated external beam radiotherapy (EBRT) delivered in 1.8–2 Gy per fraction or stereotactic radiosurgery (SRS) delivered using high dose per fraction stereotactic techniques. There are good rationale and data to support adjuvant radiation following STR of atypical meningioma,^14^ though there are data to suggest tumors with spontaneous necrosis see less benefit.^15^

One of the largest retrospective analyses of the role of RT in meningioma management was a study of 213 meningioma patients from the University of California San Francisco, that included 104 STRs—of the patients that did not receive postoperative RT, the recurrence rate was 74% as compared to 29% in the upfront irradiation group.^16^ A more recent retrospective study of 2515 atypical meningiomas identified from The National Cancer Database found adjuvant RT significantly improved overall survival (OS) compared with no adjuvant RT (hazard ratio [HR] 0.59).^17^ Other studies cite 5 year progression-free rates of 77%–88% in the RT group, as opposed to 43%–59% in the group not radiated.^18–20^ However, the benefit of adjuvant radiation following GTR is less clear, with many large retrospective studies showing conflicting findings—with some demonstrating benefit of early postoperative upfront RT^20,21^ and others not.^17,22–24^

Anaplastic meningiomas are often treated with irradiation regardless of extent of resection, given their aggressive nature and potential for rapid, early progression.^25,26^

We report the outcomes of newly diagnosed atypical meningiomas, in the context of upfront RT, at Weill Cornell Medicine over a 16-year period.

## Materials and Methods

Atypical meningiomas managed at Weill Cornell Medicine between the years 2004 and 2020 were reviewed on an institutional IRB-approved protocol for demographics, date of initial surgery, extent of resection including GTR and STR (with GTR encompassing Simpson I–III), histopathologic features, presence and type of radiation treatment, and date of first progression on MRI.

Only new diagnoses of atypical meningioma were assessed. Cases where date of initial diagnosis and first progression could not be determined because of inadequate records were excluded. Cases with only computed tomography imaging were also excluded. Primary outcomes were survival and disease progression. Pathology classification was by WHO classification at time of surgery—specimens predating WHO 2016 were not reinterpreted under the new schema.

Data analysis was done in R, with results represented by Cox proportional hazards and Kaplan–Meier survival curves. OS and progression-free survival (PFS) curves were estimated by Kaplan–Meier and compared with 2-sided log-rank test. All graphics were created in R version 4.0.3^27^ with the ggkm^28^ and survminer packages.^29^

## Results

Of the 120 new atypical meningioma cases identified in the pathology case records, 30 were excluded from analysis for inadequate records.

Ninety cases in total were analyzed, of which 56% were women (Table 1). Median age at diagnosis was 61 years of age (range 21–96). Median follow-up was 41 months (range <1 to 192 months). Median time elapsed from surgery to RT completion was 13 weeks.

**Table 1.** Characteristics of All Cases

Parameter		All (*n* = 90)
Age at diagnosis, years, median (IQR)		61 (29)
Female, *n* (%)		50 (56%)
Follow-up, months, median (IQR)		41 (45)
Presenting symptoms, *n* (%)		
Headache		27 (30%)
Motor		27 (30%)
Seizure		17 (19%)
Cognitive		15 (17%)
Vision changes		13 (14%)
Sensory		12 (13%)
Auditory changes		2 (2%)
Light-headedness		1 (1%)
Syncope		1 (1%)
Proptosis		1 (1%)
Asymptomatic		11 (12%)
Tumor location, *n* (%)		
Convexity/parasagittal	56 (62%)	Convexity (*n* = 31), parasagittal (25)
Other	34 (38%)	Sphenoid wing (*n* = 17), sella (3), anterior skull base (3), posterior fossa (4), foramen magnum (2), tentorial (5)
Gross total resection (GTR), *n* (%)		60 (67%)
Subtotal resection (STR), *n* (%)		30 (33%)

IQR, interquartile range.

**Figure 1.**
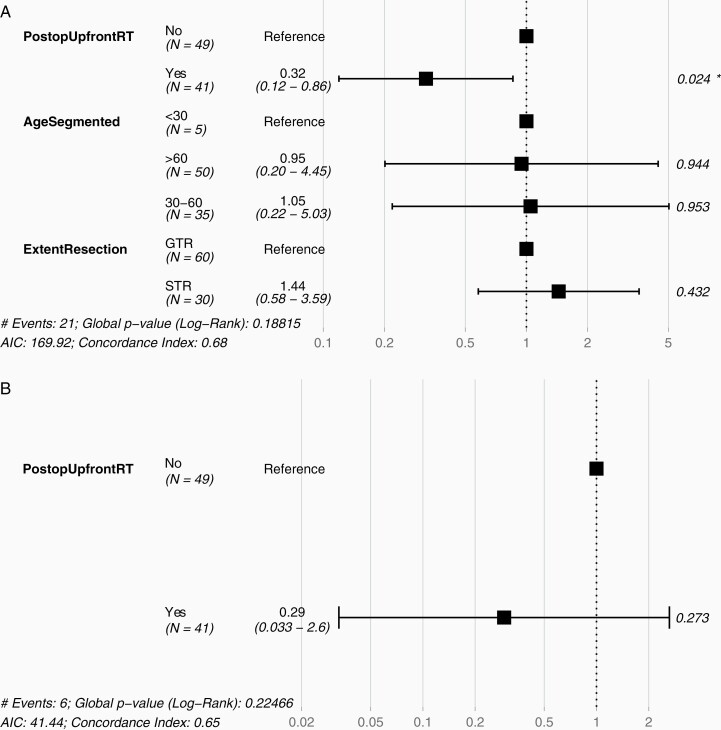
Hazard ratio for progression or death in atypical meningioma. Across all patients with newly diagnosed atypical meningioma following GTR or STR, postoperative upfront RT was associated with (A) reduced HR of progression, (B) but not of death. GTR, gross total resection; HR, hazard ratio; RT, radiotherapy; STR, subtotal resection.

Taking all 90 cases as a whole (including patients who had either GTR or STR), adjuvant RT was associated with significant reduced risk of progression (HR 0.32; 95% confidence interval [CI] 0.12–0.86; *P* = .024; Figure 1A), but no effect was seen on survival (Figure 1B).

### Gross Total Resection

Sixty cases with new diagnosis of atypical meningioma had GTR. Of these, 43% (26) had postoperative upfront RT. Sixteen patients received EBRT, from 52.2 to 60 Gy delivered in 29 to 33 fractions. Ten patients received SRS. SRS was delivered variously using Varian iX (33 Gy in 3 fractions, 25 Gy in 5 fractions), Varian TrueBeam (33 Gy in 3 fractions), Novalis (24 Gy in 3 fractions, 25 Gy in 5 fractions), and CyberKnife (25 Gy in 5 fractions to 81% isodose line). The equivalent dose in 2 Gy per fraction (EQD2) is 75.43 Gy for 33 Gy in 3 fractions, 44.57 Gy for 24 Gy in 3 fractions, and 35.71 Gy for 25 Gy in 5 fractions (assuming alpha/beta ratio 5). The remainder were observed postoperatively. There was no statistically significant difference in age at diagnosis, follow-up duration, Simpson grading, tumor location, and mitotic count between the postoperative upfront RT group versus the observation only group (Table 2).

**Table 2.** Characteristics of Gross Total Resected Cases

Gross Total Resection for Newly Diagnosed Atypical Meningioma	Postoperative Upfront RT (*n* = 26)	Postoperative Observation (*n* = 34)	
Age at diagnosis, median, years (IQR)	60.6 (24)	61.1 (32)	*P* = .90
Female, *n* (%)	15 (58%)	19 (56%)	
Follow-up, median, months (IQR)	37 (38)	44 (39)	*P* = .57
Simpson			
I	23	23	χ ^2^*P* = .10
II	3	4	
III	0	4	
Unspecified	0	3	
Location			
Convexity/parasagittal	19	22	χ ^2^*P* = .68
Other	7	12	
Mitotic count, median (IQR)	5 (2)	4 (2)	*P* = .79
EBRT regimens, total dose/fractions (*n*)	52.2 Gy/29 fx (1); 54 Gy/30 fx (10); 59.4 Gy/33 fx (2); 60 Gy/30 fx (2); unspecified (1)		
SRS regimens, total dose/fractions (*n*)	24 Gy/3 fx (1); 25 Gy/5 fx (7); 33 Gy/3 fx (2)		

No statistically significant difference between the 2 groups in age, median follow-up, Simpson grade, location (convexity/parasagittal vs all other locations), or mitoses. “Other” locations include anterior skull base, clivus, foramen magnum, posterior fossa, sella, sphenoid wing, and tentorium. EBRT, external beam radiotherapy; IQR, interquartile range; RT, radiotherapy; SRS, stereotactic radiosurgery.

**Figure 2.**
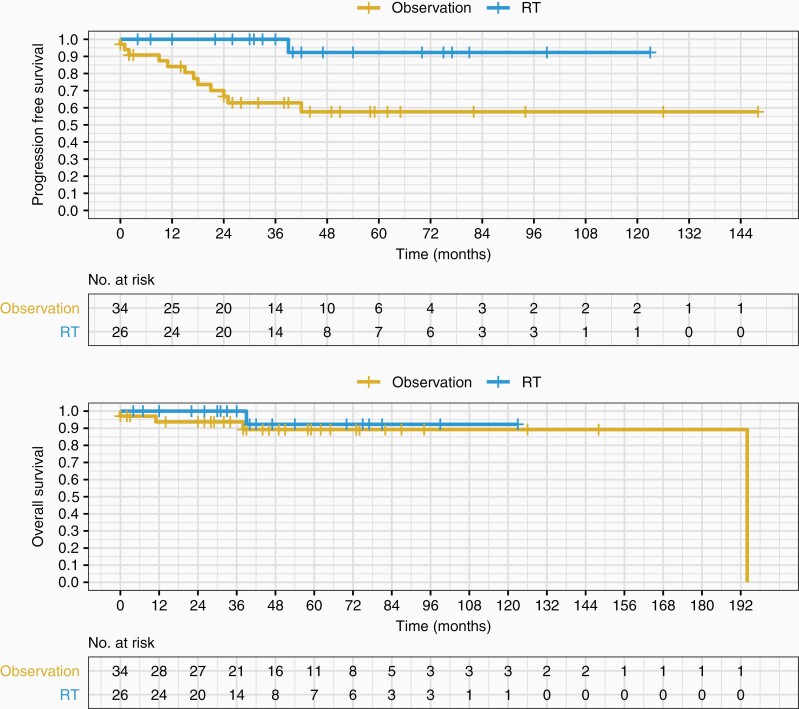
PFS and OS of observation versus upfront RT following GTR. Postoperative upfront RT (EBRT or SRS) following GTR in newly diagnosed atypical meningioma is associated with improved PFS compared to postoperative observation alone (log-rank *P* = .003). No improvement in OS with RT post-op compared to observation alone post-op (log-rank *P* = .5). EBRT, external beam radiotherapy; GTR, gross total resection; OS, overall survival; PFS, progression-free survival; RT, radiotherapy; SRS, stereotactic radiosurgery.

Postoperative upfront RT was associated with superior PFS as compared to observation alone (log-rank *P* value .003, Figure 2A). PFS at 12 months (PFS12) and at 36 months (PFS36) was superior with the upfront RT arm compared to the observation arm (100% vs 84% at 12 months, and 100% vs 63% at 36 months, respectively). HR of tumor progression or death was 0.09 with postoperative upfront RT compared to postoperative observation (95% CI 0.01–0.68; *P* = .02). However, HR was not significant for OS (HR 0.46; 95% CI 0.05–4.45; *P* = .50; Figure 2B). OS at 36 months (OS36) was 100% in the upfront RT group, and 94% in the observation group.

Of the 26 patients receiving postoperative upfront RT after GTR, no patients progressed during follow-up. Of the 34 patients who were observed and did not receive RT after GTR, 29% (10 of 34 cases) progressed at a median of 17.5 months. Of the 10 progressors, 2 were lost to follow-up, 2 were treated with both surgical re-resection and salvage RT, 1 was treated with salvage surgery alone, and 5 were treated with salvage RT.

### Subtotal Resection

A total of 30 patients had a STR for newly diagnosed atypical meningioma. Of these, 50% (15 of 30) received postoperative upfront RT while the others were observed only (Table 3). SRS was delivered variously using Varian iX (33 Gy in 3 fractions) and Novalis (25 Gy in 5 fractions). The EQD2 is 75.43 Gy for 33 Gy in 3 fractions, and 35.71 Gy for 25 Gy in 5 fractions (for alpha/beta ratio 5).

**Table 3.** Characteristics of Subtotally Resected Cases

Subtotal Resection for Newly Diagnosed Atypical Meningioma	Postoperative Upfront RT (*n* = 15)	Postoperative Observation (*n* = 15)	
Age at diagnosis, median, years (IQR)	56 (17)	73 (19)	*P* = .08
Female, *n* (%)	9 (60%)	7 (47%)	
Follow-up, median, months (IQR)	65 (56)	39 (42)	*P* = .35
Simpson			
IV	15	15	
V	0	0	
Location			
Convexity/parasagittal	7	8	χ ^2^*P* = 1
Other	8	7	
Mitotic count, median (IQR)	4 (0)	6 (3)	*P* = .1
EBRT regimens, total dose/fractions (*n*)	50.4 Gy/28 fx (1); 54 Gy/30 fx (2); 59.4 Gy/33 fx (3); 60 Gy/30 fx (2); 60.4 Gy/33 fx (1); unspecified (1)		
SRS regimens, total dose/fractions (*n*)	25 Gy/5 fx (2); 30 Gy/6 fx (1); 33 Gy/3 fx (2)		

No statistically significant differences between upfront and non-upfront RT cases, although median age of non-RT group was higher. “Other” locations include meningiomas in anterior skull base, foramen magnum, posterior fossa, sella, sphenoid wing, and tentorium. EBRT, external beam radiotherapy; IQR, interquartile range; RT, radiotherapy; SRS, stereotactic radiosurgery.

**Figure 3.**
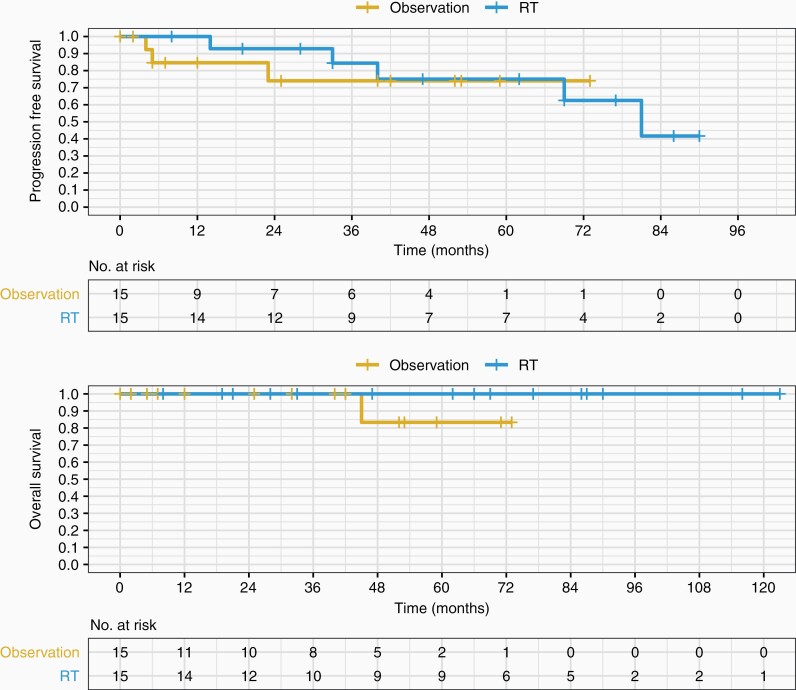
PFS and OS of observation versus upfront RT following STR. Postoperative upfront RT (EBRT or SRS) following STR in newly diagnosed atypical meningioma is not statistically significant by PFS nor OS. EBRT, external beam radiotherapy; OS, overall survival; PFS, progressionfree survival; RT, radiotherapy; SRS, stereotactic radiosurgery; STR, subtotal resection.

In patients who had STR, there was no significant difference in PFS (Figure 3A) or OS (Figure 3B) between the patients who had postoperative upfront RT versus those who were monitored (log-rank PFS *P* = .73; log-rank OS *P* = 1; HR 0.76 for progression or death with 95% CI 0.16–3.5, *P* = .73). Nevertheless, PFS at 12 and 36 months was better in the postoperative upfront RT group compared to postoperative observation alone—PFS12 100% compared to 85% for observation, PFS36 84% compared to 74% for observation. OS36 was 100% in both arms. Median time to progression was also longer in the postoperative upfront RT group as compared to the postoperative observation group—40 months (mean 47 months; range 14–81 months) and 5 months (mean 11 months; range 4–23 months), respectively (*P* = .04).

Of the STR patients that received postoperative RT, 5 patients progressed—only one received SRS (the other 4 received EBRT). Median time to progression in the EBRT group was 51 months (range 14–81 months); time to progression for the lone SRS progressor was 40 months. No statistically significant difference in PFS was found between SRS and EBRT (log-rank *P* = .4). There were no deaths in any of the STR patients receiving postoperative RT (either SRS or EBRT).

Three patients in the observation arm progressed. All 3 of these progressors were treated with salvage RT.

## Discussion

Atypical meningioma occupies a middle position between the indolent WHO Grade I meningioma and the aggressive anaplastic meningioma, and encompasses tumors with a wide range of clinical behaviors. Management of gross totally resected atypical meningioma remains controversial.

This study retrospectively assessed the effect of postoperative upfront RT on outcomes in newly diagnosed atypical meningioma. The practice pattern in our cohort showed about 46% of patients received RT (43% for GTR, 50% for STR). In patients who had GTR, postoperative upfront RT was associated with superior PFS at 12 and 36 months compared to observation, and HR for PFS was 0.09 (95% CI 0.01–0.68; *P* = .02). HR for OS did not reach significance, though OS at 12 and 36 months was superior in the postoperative upfront RT group. In patients who had STR, PFS at 12 and 36 months was numerically superior for the patients who had postoperative upfront RT compared to those who were observed, though HR for progression or death was not significant. Multiple prior studies have demonstrated improved outcomes with postoperative upfront RT following STR, and the failure to replicate those results in our dataset likely reflects lack of sufficient power in the STRs group.

It is worth noting that there has been a substantial increase in the use of SRS^30^ for the treatment of meningioma in recent years, likely representing its increased convenience to patients, improving SRS techniques/capability, and the potential for decreased toxicity with improved cognitive outcomes.

Limitations of our study include its single-center and retrospective nature as well as patients lost to follow-up without serial imaging. Pathology was not reinterpreted based on changes made to the atypical meningioma classification in WHO 2016 (namely, brain invasion). The associations with outcomes identified in the study also may be confounded by clinical factors at point of care that are not apparent retrospectively during chart review. This is especially the case with patients with subtotally resected tumors, a population which has the potential to be much more heterogeneous, with many more confounding clinical factors that may affect the decision of whether to treat with adjuvant radiation. The group who did not receive postoperative RT had a much higher median age than the radiated group. While this did not reach statistical significance, the age difference spanned nearly 2 decades. In practice, factors such as age—a surrogate for life expectancy, comorbid conditions, and functional status—are taken into consideration in selecting cases for RT, and are difficult to extricate from the effect of RT itself on outcomes. Atypical meningioma patients who have had GTRs are may also exhibit these confounding factors.

In general, atypical meningiomas are inherently challenging to study because of their much smaller numbers compared to WHO Grade I meningiomas and protracted clinical course. Despite review of over 15 years-worth of retrospective data at a busy urban tertiary academic medical center, there were significant obstacles to data integrity because of the long follow-up course of atypical meningiomas. A significant number of cases were referred in for second or third recurrence, and did not have adequate original outside records for the purpose of this study, which limited the number of cases that could be analyzed. Furthermore, patients who were clinically stable had a tendency to discontinue follow-up within several years, likely due to lack of desire to continue indefinitely with clinical visits and MRIs. Social factors, such as relocation outside the metropolitan area or changes in health insurance policy, may also have played a role in limiting individual follow-up. Although the longest follow-up for all cases was 192 months, such extended follow-up was rare, with median follow-up of only 41 months. A longer follow-up period may have captured more progressions, particularly given that median time to progression for the postoperative upfront RT groups approached time for median follow-up (40 months).

Prospective multicenter clinical trials, such as the ongoing NRG-BN003 or ROAM/EORTC-1308,^31^ have the potential to circumvent many of these issues, with both incentive and resources directed at retention—however the expense in coordinating such multiyear multicenter efforts is high.

Future improvements in the management of atypical meningiomas will be dependent on a better molecular understanding of these tumors and clearer patient stratification in this heterogeneous space. DNA methylation-based classifiers promise to supplement histologic classification in predicting prognosis and outcomes and creating more accurate patient groups.^32^ Additionally, radiotracers such as gallium-68 (^68^Ga)-labeled dodecanetetraacetic acid-tyrosine-3-octreotate (DOTATATE) and DOTA-(Tyr3)-octreotide (DOTATOC) have may better delineating the extent of meningiomas for RT planning, and for discriminating meningioma from necrosis or nontumor tissue.^33,34^ DOTATATE may improve assessment of disease extent particularly in recurrent or residual disease.^35^ Dynamic contrast imaging has also been explored as a potential biomarker in stratification of meningiomas.^36^

In conclusion, the role of adjuvant RT in the context of the gross totally resected atypical meningioma remains contentious. This retrospective analysis of newly diagnosed atypical meningiomas at a tertiary academic medical center identified an association of postoperative upfront RT with prolonged PFS in patient who had GTR. OS was also numerically superior at 12 and 36 months however the HR was not significant. In the group of patients who had STRs, PFS was numerically superior at 12 and 36 months in the postoperative upfront RT group, but HR for progression or death was not statistically significant. At less than half the size of the GTR group, this STR portion of the analysis may have been underpowered. The long follow-up course for atypical meningioma renders it an especially difficult entity to study. Individual clinical factors are likely to continue to drive the decision for adjuvant RT, particularly for STRs. Multicenter prospective randomized studies such as ROAM/EORTC-1308 and NRG-BN003 (both already underway) will be important in providing more definitive answers about the role of adjuvant RT in gross totally resected atypical meningiomas.

## Notes

**Authorship Statement.** R.R. conceived the study. Data collection was performed by P.C.P. Analysis and interpretation of the data were done by R.R., P.C.P., T.H.S., S.C.P., G.C.C., J.I., and H.A.F. The manuscript was drafted by P.C.P. and R.R. All authors participated in revising and approving the final manuscript.
